# Isopropyl 2-(5-bromo-3-methyl­sulfinyl-1-benzofuran-2-yl)acetate

**DOI:** 10.1107/S160053680803506X

**Published:** 2008-11-08

**Authors:** Hong Dae Choi, Pil Ja Seo, Byeng Wha Son, Uk Lee

**Affiliations:** a Department of Chemistry, Dongeui University, San 24 Kaya-dong, Busanjin-gu, Busan 614-714, Republic of Korea; b Department of Chemistry, Pukyong National University, 599-1 Daeyeon 3-dong, Nam-gu, Busan 608-737, Republic of Korea

## Abstract

In the title compound, C_14_H_15_BrO_4_S, the O atom and the methyl group of the methyl­sulfinyl substituent lie on opposite sides of the plane of the benzofuran fragment. The crystal structure is stabilized by C—H⋯π inter­actions between a methyl H atom and the benzene ring of a neighbouring mol­ecule, and by weak inter­molecular C—H⋯O hydrogen bonds.

## Related literature

For the crystal structures of similar alkyl 2-(3-methyl­sulfinyl-1-benzofuran-2-yl)acetate derivatives, see: Choi *et al.* (2007 ▶, 2008 ▶). 
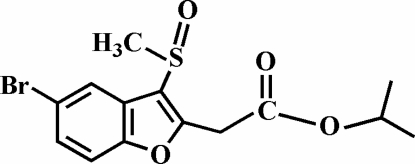


## Experimental

### 

#### Crystal data

C_14_H_15_BrO_4_S
*M* *_r_* = 359.23
Triclinic, 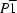

*a* = 7.947 (1) Å
*b* = 10.078 (1) Å
*c* = 10.868 (1) Å
α = 69.623 (2)°
β = 82.027 (2)°
γ = 67.409 (2)°
*V* = 753.33 (14) Å^3^
*Z* = 2
Mo *K*α radiation
μ = 2.88 mm^−1^
*T* = 298 (2) K
0.40 × 0.40 × 0.20 mm

#### Data collection

Bruker SMART CCD diffractometer
Absorption correction: multi-scan (*SADABS*; Sheldrick, 1999 ▶) *T*_min_ = 0.321, *T*_max_ = 0.559
3979 measured reflections
2604 independent reflections
2123 reflections with *I* > 2σ(*I*)
*R*_int_ = 0.011

#### Refinement

*R*[*F* ^2^ > 2σ(*F* ^2^)] = 0.031
*wR*(*F* ^2^) = 0.085
*S* = 1.02
2604 reflections
181 parameters
H-atom parameters constrained
Δρ_max_ = 0.46 e Å^−3^
Δρ_min_ = −0.46 e Å^−3^

### 

Data collection: *SMART* (Bruker, 2001 ▶); cell refinement: *SAINT* (Bruker, 2001 ▶); data reduction: *SAINT*; program(s) used to solve structure: *SHELXS97* (Sheldrick, 2008 ▶); program(s) used to refine structure: *SHELXL97* (Sheldrick, 2008 ▶); molecular graphics: *ORTEP-3* (Farrugia, 1997 ▶) and *DIAMOND* (Brandenburg, 1998 ▶); software used to prepare material for publication: *SHELXL97*.

## Supplementary Material

Crystal structure: contains datablocks global, I. DOI: 10.1107/S160053680803506X/sg2276sup1.cif

Structure factors: contains datablocks I. DOI: 10.1107/S160053680803506X/sg2276Isup2.hkl

Additional supplementary materials:  crystallographic information; 3D view; checkCIF report

## Figures and Tables

**Table 1:** Hydrogen-bond geometry (Å, °)

*D*—H⋯*A*	*D*—H	H⋯*A*	*D*⋯*A*	*D*—H⋯*A*
C3—H3⋯O2^i^	0.93	2.50	3.392 (4)	160
C10—H10*B*⋯O2^ii^	0.97	2.40	3.365 (4)	173
C13—H13*C*⋯*Cg*^iii^	0.96	2.78	3.526 (4)	136

Symmetry codes: (i) 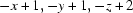; (ii) 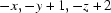; (iii) 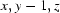. *Cg* is the centroid of the C2–C7 benzene ring.

**Fig. 1.**
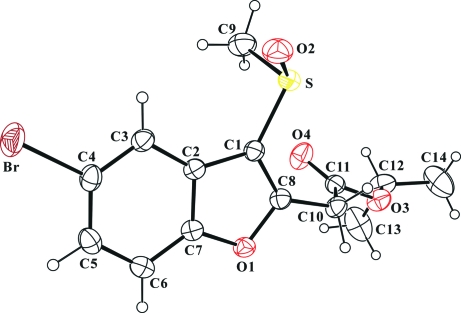
The molecular structure of the title compound, showing displacement ellipsoids drawn at the 30% probability level.

**Fig. 2.**
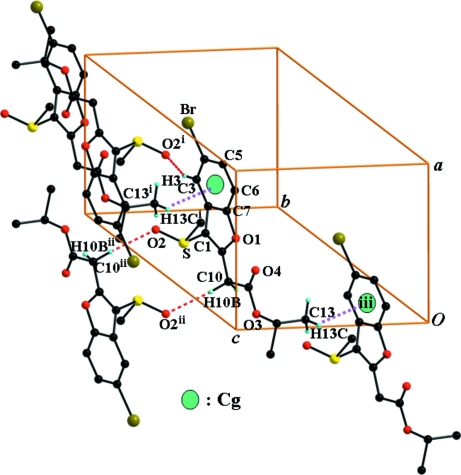
C—H···π and C—H···O interactions (dotted lines) in the title compound. Cg denotes ring centroid. [Symmetry code: (i) x, y + 1, z; (ii) -x, -y + 1, -z + 2; (iii) x, y - 1, z.]

## supplementary crystallographic information

### Comment

This work is related to our previous communications on the synthesis and structure of alkyl 2-(3-methylsulfinyl-1-benzofuran-2-yl)acetate analogues, *viz*. ethyl 2-(5-bromo-3-methylsulfinyl-1-benzofuran-2-yl)acetate (Choi *et al.*, 2007) and isopropyl 2-(5-methyl-3-methylsulfinyl-1-benzofuran-2-yl) acetate (Choi *et al.*, 2008). Here we report the crystal structure of the title compound, isopropyl 2-(5-bromo-3-methylsulfinyl-1-benzofuran-2-yl) acetate (Fig. 1).

The benzofuran unit is essentially planar, with a mean deviation of 0.014 (2) Å from the least-squares plane defined by the nine constituent atoms. The molecular packing (Fig. 2) is stabilized by intermolecular C—H···π interactions between a methyl H atom of isoproyl group and the benzene ring of the benzofuran fragment, with a C13—H13C···*Cg*^iii^ separation of 2.78 Å (Fig. 2 and Table 1; *Cg* is the centroid of the C2–C7 benzene ring, symmetry code as in Fig. 2). The molecular packing is further stabilized by two intermolecular C—H···O hydrogen bonds (Fig. 2 and Table 1)

### Experimental

77% 3-chloroperoxybenzoic acid (370 mg, 1.65 mmol) was added in small portions to a stirred solution of isopropyl 2-(5-bromo-3-methylsulfanyl-1-benzofuran-2-yl)acetate (515 mg, 1.50 mmol) in dichloromethane (40 ml) at 273 K. After being stirred for 3 h at room temperature, the mixture was washed with saturated sodium bicarbonate solution and the organic layer was separated, dried over magnesium sulfate, filtered and concentrated in vacuum. The residue was purified by column chromatography (hexane-ethyl acetate, 1:2 *v*/*v*) to afford the title compound as a colorless solid [yield 78%, m.p. 429–430 K; *R*_f_ = 0.74 (hexane-ethyl acetate, 1;2 *v*/*v*)]. Single crystals suitable for X-ray diffraction were prepared by evaporation of a solution of the title compound in benzene at room temperature. Spectroscopic analysis: ^1^H NMR (CDCl_3_, 400 MHz) δ 1.26 (d, J = 6.24 Hz, 6H), 3.07 (s, 3H), 4.01 (s, 2H), 5.01–5.08 (m, 1H), 7.39 (d, J = 8.80 Hz, 1H), 7.48 (d, J = 8.76 Hz, 1H), 8.09 (s, 1H); EI—MS 360 [*M*+2], 358 [*M*^+^].

### Refinement

All H atoms were geometrically positioned and refined using a riding model, with C—H = 0.98 (methine), 0.93 (aromatic), 0.97 (methylene), and 0.96 Å (methyl) H atoms, respectively, and with *U*_iso_(H) = 1.2Ueq(C) (aromatic, methylene & methine), and 1.5Ueq(C) (methyl) H atoms.

### Crystal data

**Table:**

C_14_H_15_BrO_4_S	*Z* = 2
*M**_r_* = 359.23	*F*_000_ = 364
Triclinic, *P*1	*D*_x_ = 1.584 Mg m^−^^3^
Hall symbol: -P_1	Melting point = 429–430 K
*a* = 7.947 (1) Å	Mo *K*α radiation λ = 0.71073 Å
*b* = 10.078 (1) Å	Cell parameters from 2313 reflections
*c* = 10.868 (1) Å	θ = 2.8–28.2º
α = 69.623 (2)º	µ = 2.88 mm^−^^1^
β = 82.027 (2)º	*T* = 298 (2) K
γ = 67.409 (2)º	Block, colorless
*V* = 753.33 (14) Å^3^	0.40 × 0.40 × 0.20 mm

### Data collection

**Table:**

Bruker SMART CCD diffractometer	2604 independent reflections
Radiation source: fine-focus sealed tube	2123 reflections with *I* > 2σ(*I*)
Monochromator: graphite	*R*_int_ = 0.011
Detector resolution: 10.0 pixels mm^-1^	θ_max_ = 25.0º
*T* = 298(2) K	θ_min_ = 2.3º
φ and ω scans	*h* = −9→5
Absorption correction: multi-scan(SADABS; Sheldrick, 1999)	*k* = −11→11
*T*_min_ = 0.321, *T*_max_ = 0.559	*l* = −12→12
3979 measured reflections	

### Refinement

**Table:**

Refinement on *F*^2^	Secondary atom site location: difference Fourier map
Least-squares matrix: full	Hydrogen site location: difference Fourier map
*R*[*F*^2^ > 2σ(*F*^2^)] = 0.031	H-atom parameters constrained
*wR*(*F*^2^) = 0.085	*w* = 1/[σ^2^(*F*_o_^2^) + (0.042*P*)^2^ + 0.4644*P*] where *P* = (*F*_o_^2^ + 2*F*_c_^2^)/3
*S* = 1.02	(Δ/σ)_max_ < 0.001
2604 reflections	Δρ_max_ = 0.46 e Å^−^^3^
181 parameters	Δρ_min_ = −0.46 e Å^−^^3^
Primary atom site location: structure-invariant direct methods	Extinction correction: none

### Special details

**Table:**

Geometry. All e.s.d.'s (except the e.s.d. in the dihedral angle between two l.s. planes) are estimated using the full covariance matrix. The cell e.s.d.'s are taken into account individually in the estimation of e.s.d.'s in distances, angles and torsion angles; correlations between e.s.d.'s in cell parameters are only used when they are defined by crystal symmetry. An approximate (isotropic) treatment of cell e.s.d.'s is used for estimating e.s.d.'s involving l.s. planes.
Refinement. Refinement of *F*^2^ against ALL reflections. The weighted *R*-factor *wR* and goodness of fit *S* are based on *F*^2^, conventional *R*-factors *R* are based on *F*, with *F* set to zero for negative *F*^2^. The threshold expression of *F*^2^ > σ(*F*^2^) is used only for calculating *R*-factors(gt) *etc*. and is not relevant to the choice of reflections for refinement. *R*-factors based on *F*^2^ are statistically about twice as large as those based on *F*, and *R*- factors based on ALL data will be even larger.

### Fractional atomic coordinates and isotropic or equivalent isotropic displacement parameters (Å^2^)

**Table:**

	*x*	*y*	*z*	*U*_iso_*/*U*_eq_	
Br	0.70616 (5)	0.79293 (4)	0.62791 (4)	0.07083 (16)	
S	0.25401 (10)	0.39786 (9)	0.96079 (7)	0.0469 (2)	
O1	0.1648 (2)	0.5361 (2)	0.58148 (17)	0.0407 (4)	
O2	0.2529 (3)	0.5174 (3)	1.0122 (2)	0.0624 (6)	
O3	−0.0241 (3)	0.1540 (2)	0.7747 (2)	0.0532 (5)	
O4	0.2603 (3)	0.1418 (3)	0.7888 (3)	0.0651 (6)	
C1	0.2522 (4)	0.4734 (3)	0.7885 (3)	0.0377 (6)	
C2	0.3526 (3)	0.5637 (3)	0.7033 (3)	0.0367 (6)	
C3	0.4811 (4)	0.6187 (3)	0.7202 (3)	0.0422 (6)	
H3	0.5264	0.5956	0.8026	0.051*	
C4	0.5377 (4)	0.7090 (3)	0.6087 (3)	0.0457 (7)	
C5	0.4752 (4)	0.7448 (3)	0.4839 (3)	0.0487 (7)	
H5	0.5184	0.8063	0.4119	0.058*	
C6	0.3495 (4)	0.6897 (3)	0.4662 (3)	0.0441 (7)	
H6	0.3065	0.7116	0.3834	0.053*	
C7	0.2906 (4)	0.6004 (3)	0.5777 (3)	0.0377 (6)	
C8	0.1423 (4)	0.4614 (3)	0.7115 (3)	0.0389 (6)	
C9	0.4813 (5)	0.2606 (4)	0.9795 (3)	0.0648 (9)	
H9A	0.5068	0.2067	1.0711	0.097*	
H9B	0.5660	0.3113	0.9426	0.097*	
H9C	0.4930	0.1903	0.9350	0.097*	
C10	0.0116 (4)	0.3821 (3)	0.7411 (3)	0.0437 (7)	
H10A	−0.0696	0.4239	0.6671	0.052*	
H10B	−0.0617	0.4014	0.8165	0.052*	
C11	0.1016 (4)	0.2131 (3)	0.7692 (3)	0.0416 (6)	
C12	0.0375 (5)	−0.0102 (4)	0.7976 (4)	0.0630 (9)	
H12	0.1468	−0.0652	0.8524	0.076*	
C13	0.0742 (7)	−0.0350 (4)	0.6682 (5)	0.0978 (16)	
H13A	0.1649	0.0055	0.6220	0.117*	
H13B	−0.0361	0.0153	0.6180	0.117*	
H13C	0.1175	−0.1418	0.6810	0.117*	
C14	−0.1201 (8)	−0.0562 (5)	0.8665 (4)	0.1008 (16)	
H14A	−0.2254	−0.0017	0.8110	0.121*	
H14B	−0.1470	−0.0329	0.9471	0.121*	
H14C	−0.0881	−0.1633	0.8851	0.121*	

### Atomic displacement parameters (Å^2^)

**Table:**

	*U*^11^	*U*^22^	*U*^33^	*U*^12^	*U*^13^	*U*^23^
Br	0.0636 (2)	0.0633 (2)	0.0936 (3)	−0.04221 (19)	−0.00607 (19)	−0.0111 (2)
S	0.0499 (4)	0.0555 (5)	0.0372 (4)	−0.0265 (4)	−0.0032 (3)	−0.0078 (3)
O1	0.0454 (11)	0.0435 (11)	0.0376 (10)	−0.0190 (9)	−0.0054 (8)	−0.0131 (9)
O2	0.0709 (15)	0.0739 (16)	0.0505 (13)	−0.0259 (12)	−0.0048 (11)	−0.0285 (12)
O3	0.0584 (13)	0.0409 (11)	0.0657 (14)	−0.0251 (10)	−0.0142 (10)	−0.0103 (10)
O4	0.0492 (14)	0.0484 (13)	0.0931 (18)	−0.0181 (11)	−0.0084 (12)	−0.0138 (12)
C1	0.0402 (15)	0.0386 (15)	0.0379 (15)	−0.0181 (12)	−0.0022 (12)	−0.0113 (12)
C2	0.0382 (14)	0.0316 (14)	0.0398 (15)	−0.0117 (11)	−0.0041 (11)	−0.0105 (11)
C3	0.0416 (15)	0.0407 (15)	0.0462 (16)	−0.0164 (13)	−0.0063 (12)	−0.0123 (13)
C4	0.0419 (16)	0.0372 (15)	0.0593 (19)	−0.0181 (13)	0.0002 (13)	−0.0131 (14)
C5	0.0498 (17)	0.0399 (16)	0.0483 (17)	−0.0164 (14)	0.0071 (14)	−0.0075 (13)
C6	0.0486 (17)	0.0403 (16)	0.0373 (15)	−0.0116 (13)	−0.0008 (12)	−0.0103 (12)
C7	0.0403 (15)	0.0327 (14)	0.0400 (15)	−0.0114 (12)	−0.0035 (12)	−0.0125 (12)
C8	0.0414 (15)	0.0372 (14)	0.0405 (15)	−0.0154 (12)	−0.0024 (12)	−0.0132 (12)
C9	0.067 (2)	0.060 (2)	0.058 (2)	−0.0173 (18)	−0.0160 (17)	−0.0083 (17)
C10	0.0425 (16)	0.0459 (16)	0.0488 (16)	−0.0203 (13)	−0.0051 (13)	−0.0159 (13)
C11	0.0480 (18)	0.0459 (16)	0.0364 (15)	−0.0246 (14)	−0.0020 (12)	−0.0106 (12)
C12	0.078 (2)	0.0388 (17)	0.073 (2)	−0.0261 (17)	−0.0265 (19)	−0.0036 (16)
C13	0.119 (4)	0.051 (2)	0.111 (4)	−0.024 (2)	0.042 (3)	−0.036 (2)
C14	0.159 (5)	0.084 (3)	0.086 (3)	−0.086 (3)	0.024 (3)	−0.022 (2)

### Geometric parameters (Å, °)

**Table:**

Br—C4	1.904 (3)	C6—C7	1.380 (4)
S—O2	1.492 (2)	C6—H6	0.9300
S—C1	1.759 (3)	C8—C10	1.479 (4)
S—C9	1.790 (4)	C9—H9A	0.9600
O1—C8	1.374 (3)	C9—H9B	0.9600
O1—C7	1.375 (3)	C9—H9C	0.9600
O3—C11	1.331 (3)	C10—C11	1.506 (4)
O3—C12	1.471 (4)	C10—H10A	0.9700
O4—C11	1.193 (3)	C10—H10B	0.9700
C1—C8	1.352 (4)	C12—C13	1.483 (5)
C1—C2	1.445 (4)	C12—C14	1.513 (6)
C2—C7	1.392 (4)	C12—H12	0.9800
C2—C3	1.394 (4)	C13—H13A	0.9600
C3—C4	1.376 (4)	C13—H13B	0.9600
C3—H3	0.9300	C13—H13C	0.9600
C4—C5	1.387 (4)	C14—H14A	0.9600
C5—C6	1.378 (4)	C14—H14B	0.9600
C5—H5	0.9300	C14—H14C	0.9600
			
O2—S—C1	107.21 (13)	H9A—C9—H9B	109.5
O2—S—C9	106.82 (16)	S—C9—H9C	109.5
C1—S—C9	98.00 (15)	H9A—C9—H9C	109.5
C8—O1—C7	106.32 (19)	H9B—C9—H9C	109.5
C11—O3—C12	118.0 (2)	C8—C10—C11	113.5 (2)
C8—C1—C2	107.3 (2)	C8—C10—H10A	108.9
C8—C1—S	123.7 (2)	C11—C10—H10A	108.9
C2—C1—S	129.0 (2)	C8—C10—H10B	108.9
C7—C2—C3	119.5 (2)	C11—C10—H10B	108.9
C7—C2—C1	104.7 (2)	H10A—C10—H10B	107.7
C3—C2—C1	135.9 (2)	O4—C11—O3	125.0 (3)
C4—C3—C2	116.6 (3)	O4—C11—C10	125.3 (3)
C4—C3—H3	121.7	O3—C11—C10	109.6 (2)
C2—C3—H3	121.7	O3—C12—C13	108.1 (3)
C3—C4—C5	123.6 (3)	O3—C12—C14	105.3 (3)
C3—C4—Br	118.0 (2)	C13—C12—C14	110.8 (3)
C5—C4—Br	118.4 (2)	O3—C12—H12	110.8
C6—C5—C4	120.2 (3)	C13—C12—H12	110.8
C6—C5—H5	119.9	C14—C12—H12	110.8
C4—C5—H5	119.9	C12—C13—H13A	109.5
C5—C6—C7	116.6 (3)	C12—C13—H13B	109.5
C5—C6—H6	121.7	H13A—C13—H13B	109.5
C7—C6—H6	121.7	C12—C13—H13C	109.5
O1—C7—C6	125.7 (2)	H13A—C13—H13C	109.5
O1—C7—C2	110.7 (2)	H13B—C13—H13C	109.5
C6—C7—C2	123.6 (3)	C12—C14—H14A	109.5
C1—C8—O1	111.0 (2)	C12—C14—H14B	109.5
C1—C8—C10	132.5 (3)	H14A—C14—H14B	109.5
O1—C8—C10	116.5 (2)	C12—C14—H14C	109.5
S—C9—H9A	109.5	H14A—C14—H14C	109.5
S—C9—H9B	109.5	H14B—C14—H14C	109.5
			
O2—S—C1—C8	−134.2 (2)	C3—C2—C7—O1	−179.9 (2)
C9—S—C1—C8	115.3 (3)	C1—C2—C7—O1	−1.4 (3)
O2—S—C1—C2	41.9 (3)	C3—C2—C7—C6	0.1 (4)
C9—S—C1—C2	−68.5 (3)	C1—C2—C7—C6	178.5 (3)
C8—C1—C2—C7	0.5 (3)	C2—C1—C8—O1	0.7 (3)
S—C1—C2—C7	−176.2 (2)	S—C1—C8—O1	177.55 (18)
C8—C1—C2—C3	178.5 (3)	C2—C1—C8—C10	178.8 (3)
S—C1—C2—C3	1.8 (5)	S—C1—C8—C10	−4.3 (5)
C7—C2—C3—C4	0.6 (4)	C7—O1—C8—C1	−1.5 (3)
C1—C2—C3—C4	−177.2 (3)	C7—O1—C8—C10	180.0 (2)
C2—C3—C4—C5	−0.7 (4)	C1—C8—C10—C11	−76.4 (4)
C2—C3—C4—Br	177.7 (2)	O1—C8—C10—C11	101.6 (3)
C3—C4—C5—C6	0.1 (5)	C12—O3—C11—O4	−4.5 (4)
Br—C4—C5—C6	−178.3 (2)	C12—O3—C11—C10	178.3 (3)
C4—C5—C6—C7	0.6 (4)	C8—C10—C11—O4	12.3 (4)
C8—O1—C7—C6	−178.1 (3)	C8—C10—C11—O3	−170.5 (2)
C8—O1—C7—C2	1.8 (3)	C11—O3—C12—C13	−89.9 (4)
C5—C6—C7—O1	179.3 (3)	C11—O3—C12—C14	151.6 (3)
C5—C6—C7—C2	−0.7 (4)		

### Hydrogen-bond geometry (Å, °)

**Table:**

*D*—H···*A*	*D*—H	H···*A*	*D*···*A*	*D*—H···*A*
C3—H3···O2^i^	0.93	2.50	3.392 (4)	160
C10—H10B···O2^ii^	0.97	2.40	3.365 (4)	173
C13—H13C···Cg^iii^	0.96	2.78	3.526 (4)	136

Symmetry codes: (i) −*x*+1, −*y*+1, −*z*+2; (ii) −*x*, −*y*+1, −*z*+2; (iii) *x*, *y*−1, *z*.
